# The Association of Thyroid Nodules With Blood Trace Elements Identified in a Cross-Section Study

**DOI:** 10.3389/fnut.2022.870873

**Published:** 2022-04-28

**Authors:** Huixian Zeng, Yuanyuan Hu, Yaosheng Luo, Yanshi Ye, Cheng Song, Genfeng Yu, Zhi Chen, Siyang Liu, Yongqian Liang, Lingling Liu, Heng Wan, Jie Shen

**Affiliations:** ^1^Department of Endocrinology and Metabolism, Shunde Hospital, Southern Medical University (The First People's Hospital of Shunde Foshan), Foshan, China; ^2^Department of Endocrinology and Metabolism, The Third Affiliated Hospital of Southern Medical University, Guangzhou, China

**Keywords:** thyroid nodule, trace elements, copper, magnesium, zinc

## Abstract

**Background:**

The association between occurrence of thyroid nodules (TNs) and trace elements detectable in blood are still inconclusive. The present study sought to determine the relationship between selected trace elements and TNs in the iodine-adequate area of Guangdong, China.

**Methods:**

A total of 1,048 participants from four communities were enrolled. A number of medical checkups were conducted to collect relevant data on anthropometric parameters, blood pressure, glucose blood levels and lipid profiles, as well as data on thyroid function, presence of thyroid autoantibodies, and trace elements. Presence of TN was diagnosed by ultrasonography.

**Results:**

Of the 1048 participants (49.5 ± 14.4 years old), 543 participants (51.8%) had TNs. Serum copper, magnesium and zinc levels are associated with the presence of TNs among healthy subjects. Subjects with higher levels of zinc, magnesium and copper had 1.23-fold, 1.04-fold, and 1.007-fold increased risks of the prevalence of TNs (*P* = 0.013, 0.017, and < 0.001, resp). Compared with the first quartile of copper content in serum, participants in the fourth quartile had the highest prevalence of TNs with an odds ratio of 8.90 (95% confidence interval (CI) 5.41, 14.94) among all participants. Women in the third quartile of magnesium level had a 1.86-fold (95%CI 1.05, 3.31) risk of the prevalence of TNs. Subjects in the highest quartile of zinc level had a 1.82-fold (95%CI 1.06, 3.16) risk of the prevalence of TNs in females.

**Conclusion:**

TNs were found highly prevalent in females in the investigated population from an iodine-adequate area of Guangdong, China. The imbalance of selected trace elements (copper, magnesium and zinc) in the body is related to the presence of TNs among healthy subjects. The observed correlation of copper on TNs warrants further studies.

## Introduction

The rapid rise of thyroid nodules incidence coincides with an increase in diagnosed thyroid cancer. Thyroid nodules are increasingly diagnosed by use of ultrasound and fine-needle aspiration biopsy. In China, a high prevalence of thyroid nodules (20.4%) was reported in the TIDE study, which is within the range of 19.0 to 46.6% that has been reported in other countries (1–4). Thyroid nodules are more common in elderly persons, in females, and in individuals living in iodine-deficient geographic areas, as well as those with a history of radiation exposure (5). A higher risk of TNs is also associated with metabolic disorders (6–8).

The presence of thyroid nodules and risk of thyroid cancer has resulted in public health concerns, however, the relevant risk factors for development of TNs and of thyroid cancer need to be investigated in detail. Trace elements present in blood, such as magnesium (Mg), manganese (Mn), selenium (Se), iron (Fe), copper (Cu), and zinc (Zn) are vital for the health of mankind, even though they are typically present at low concentrations (9, 10). In contrast, environmental exposure to toxic lead (Pb) poses a substantial risk to human health (11). Whether trace element concentrations in blood affect thyroid function is still inconclusive. Results from a case-section study in Germany suggested the absence of any relationship between serum levels of Cu, Se, Zn, or iodine concentrations and thyroid hormone in adult thyroid patients (12). Nevertheless, a positive correlation of serum Mn and Fe levels with free triiodothyronine (FT3) was demonstrated in a large population-based study based on data from the 2011–2012 US National Health and Nutrition Examination Survey (NHANES) and this was observed in both males and females. Increased total thyroxine (TT4) levels were reported to be positively associated with serum Cu levels and inversely associated with serum Fe levels in the general population (13). A randomized study suggested that Zn and Mg supplementation may have beneficial effects in patients with hypothyroidism (14). In addition, two case-control studies prompted that levels of lead among hypothyroid people were significantly increased compared to the normal control subjects (15, 16). Such observations suggest that trace elements may affect thyroid function. Moreover, trace elements might be related to thyroid autoimmunity or neoformation (17–19). A case-control study suggested that low serum concentrations of Ca, Mg, Zn, Cu, and Se in combination resulted in a higher risk of the prevalence of nodular goiter in individuals living in a mildly iodine-deficiency region (20). An insufficiency or excess of certain essential chemical elements or exposure to toxic or potentially toxic elements interacts with thyroid hormones metabolism and may interfere with thyroid homeostasis (21). The oxidant and antioxidant balance of the body may be disrupted by alterations in the trace elements levels of body fluids, which may affect the endocrine system resulting in diverse thyroid disorders (16).

Research focusing on the relationship between serum trace element levels and thyroid nodules is sparse, and previous analyses were mainly based on case-control or hospital-based studies. These do not capture the association between trace elements serum levels and thyroid nodules in healthy subjects. To fill this data gap, research was conducted to identify the association of selected trace elements (Zn, Mg, Fe, Cu, Mn, and Pb) serum levels with thyroid nodules among healthy adults living in an iodine-adequate area of Guangdong, China.

## Materials and Methods

### Study Participants

**Figure 1:**
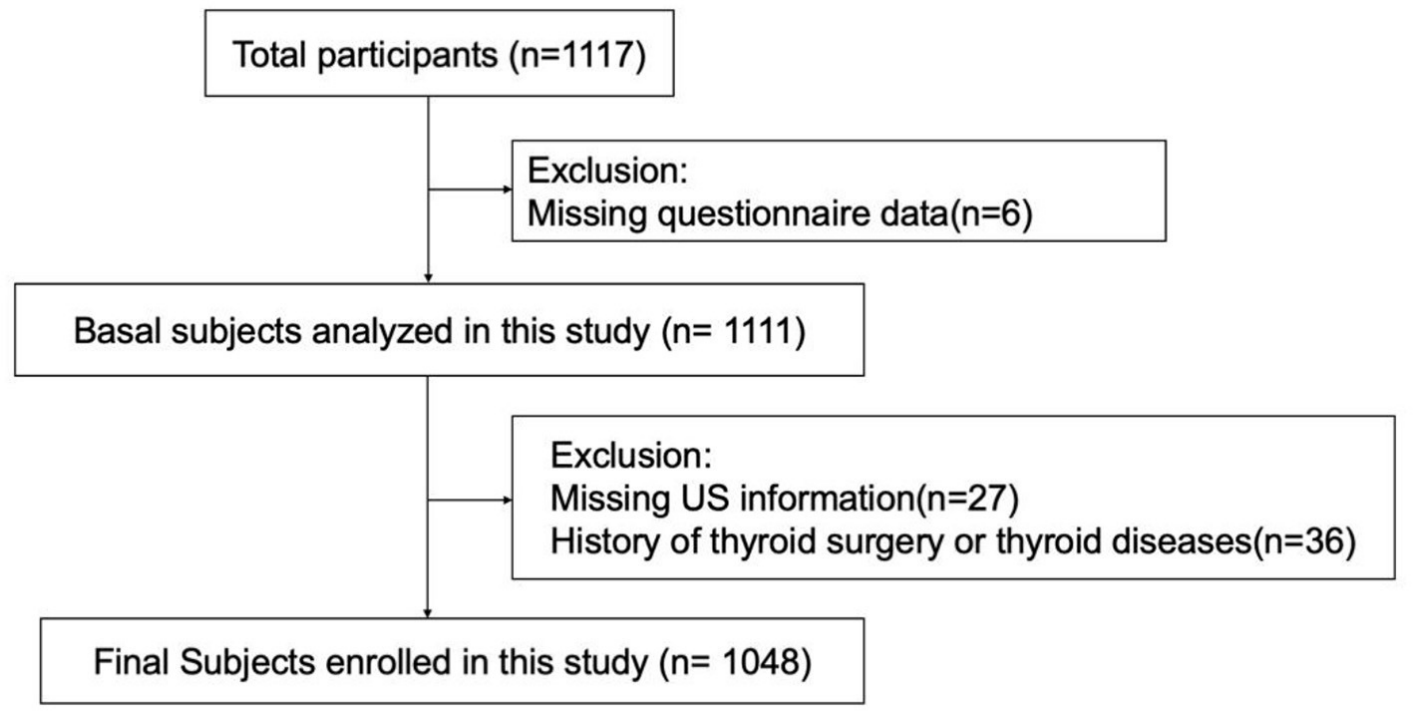
Flowchart of the inclusion and exclusion of participants.

An ongoing cross-sectional study on metabolic diseases and their risk factors in the Shunde area (www.chictr.org.cn, ChiCTR2100054130) of Guangdong, China, was employed here. A stratified cluster sampling method was applied. The enrolled study participants originated from four communities in Lecong Town, Shunde District, Foshan, China. The study was conducted in 2021. Initially, 1,117 adults (over 18 years of age) were enrolled. Participants for which questionnaire data were missing (*n* = 6) or ultrasound data was not available (*n* = 27) were excluded, as were individuals with a history of thyroid surgery or thyroid disease (*n* = 36). This left a total of 1,048 subjects who were included in the analysis (Figure 1). All participants provided signed consent after they had been fully informed about the research purpose and procedure. Ethical approval was granted from the Ethics Committee of Shunde Hospital, Southern Medical University, Shunde, Foshan, China (20211103).

### Data Collection

A standard questionnaire was used to collect sociodemographic characteristics, individual disease history, first-degree family disease history, and relevant lifestyle factors. For all individuals their weight, height and blood pressure were measured according to a standard protocol and recorded. All anthropometric and biochemical data were collected at the same time. The body mass index (BMI) was calculated from weight and length (kg/m^2^). Insulin resistance was evaluated by homeostasis model assessment of insulin resistance (HOMA-IR) using the formula HOMA-IR = [FPG (mmol/L) × FINS (IU/mL)]/22.5.

### Laboratory Assays

Serum samples were collected by venous puncture after an overnight fast between 7:00 AM and 10:00 AM. The blood samples were cooled down to 4°C and within 2–4 hours they were transported under cooling to a central laboratory that was certified by the College of American Pathologists (CAP). Spot morning urine samples were collected, the urine samples were sub-packed, coded and stored at −80°C. The following laboratory assays were performed: fasting plasma glucose (FPG) was assessed by Hitachi, LABOSPECT 008AS (Tokyo, JAPAN). Total cholesterol (TC), triglycerides (TG), low-density lipoprotein (LDL), and high-density lipoprotein (HDL) were measured by Mindray, BS-800 (Shenzhen, China). Insulin was detected by chemiluminescence (DiaSorin, LIAISON XL, Saluggia, Italy). Glycated hemoglobin (HbA1c) was assessed by high-performance liquid chromatography (TOSOH, HLC-723 G8, Tokyo, JAPAN). Thyroid peroxidase antibodies (TPOAb) were detected by chemiluminescence (SNIBE, Biolumi 8000, Shenzhen, China) and thyroglobulin antibodies (TgAb) by Beckman, UniCel DxI800, (Brea, USA). Thyroid-stimulating hormone (TSH), free triiodothyronine (FT3), and free thyroxine (FT4) levels were measured by chemiluminescent immunoassays (Siemens, Centaur XPT, Erlangen, Germany). Finally, serum concentrations of the selected trace elements Zn, Mg, Fe, Cu, Pb, and Mn were assessed by inductively coupled plasma mass spectrometry (ICP-MS) (Thermo Fisher Scientific, iCAP RQ, Waltham, USA). Urinary iodine was detected by stripping voltammetry analysis method (Shenrui SR-L100, Wuxi, China). Urine creatinine was determined by the uric dry-chemistry method. The urinary iodine excretion (UIE), expressed as the urinary iodine/creatinine (UIC/UCr) ratio.

### Thyroid Ultrasonography

Ultrasound investigations of the thyroid were performed by two registered physicians who both had a professional certificate for ultrasonography (awarded by the Ministry of Health of China). Ultrasound was performed using B-mode US imaging (MX7, Mindray Shenzhen, P.R. China) with a 13 MHz linear array probe.

### Definition

Thyroid nodules were defined to be present when sized > or equal to 2 mm in diameter. The following reference ranges for normal thyroid functions were used: 3.28–6.47 pmol/L for FT3, 7.64–16.03 pmol/L for FT4, and 0.49–4.91 μIU/mL for TSH. A value of TPOAb > 30 U/mL or of TgAb > 9 IU/mL was defined as positive. Laboratory reference ranges of the selected trace elements were following: Manganese (Mn), 6.6–21.6 μg/L; Magnesium (Mg), 26.9–49.4 mg/L; Copper (Cu), 749.3–1394.5μg/L; Zinc (Zn), 5.0–7.5 mg/L; Iron (Fe), 421.1–660.8 mg/L; Lead (Pb) < 100 μg/L. Smoking status was classified as current smokers (past consumption amounted to at least 100 cigarettes and the person was currently smoking), ex-smokers (quit smoking for more than 6 months) and non-smokers (22). Excessive alcohol consumption was defined as ≥ 210 g intake of ethanol per week for males and ≥ 140 g per week for females (23).

### Statistical Analysis

Statistical analyses were performed using IBM SPSS Statistics V.22 (IBM Corp, Armonk, NY, USA). Two-tailed *P* values below 0.05 were considered as statistically significant. Continuous variables with normal distribution were summarized as the mean±SD, and non-normally distributed variables as median and interquartile range for the 25^th^-75^th^ percentile. Categorical variables are presented as percentages. Continuous variables were compared with Student's *t*-test. The Mann-Whitney U test was applied to non-normally distributed data, and the Pearson χ^2^ test for dichotomous variables. The association of thyroid nodules and trace elements was assessed by binary logistic regression. Model 1 included sex, age and UIE. Model 2 covered sex, age, UIE, BMI, smoking status, excessive alcohol consumption, HbA1c, TC, TG and TSH. Sex-stratified analysis were further performed. The odds ratios (OR) and 95% confidence intervals (CIs) were calculated using logistic regression to assess the risk of TN for each quartile of trace elements, using the lowest quartile as the reference. The regression model was adjusted for sex, age, UIE, BMI, smoking status, excessive alcohol consumption, HbA1c, TC, TG, and TSH. The restricted cubic spline analyses for the nonlinear analysis between selected trace elements and the prevalence of TNs were completed by the statistical package R (http://www.R-project.org, The R Foundation) and Empower (R) (www.empowerstats.com; X&Y Solutions, Inc., Boston, MA, USA).

## Results

### Clinical Characteristics of Participants With and Without Thyroid Nodule

The general demographic and clinical characteristics of all 1,048 subjects (458 men and 590 women) included in the study are summarized in Table 1. The mean age was 49.5 ± 14.4 years, and the mean BMI was 24.0 ± 3.60 kg/m^2^. A total of 15.7% currently smoked and 7.2% were reported excessive alcohol consumption. Thyroid nodules (TNs) were found in 51.8% and lower in men (33.5%) than in women (66.5%). The proportion of smokers and high-risk alcohol consumers were both significantly higher in subjects without TNs than in those with TNs (*P* < 0.05). Five metabolic factors were associated with presence of TNs: a higher level of HOMA-IR, HbA1c, LDL, total cholesterol, and higher systolic pressure (all *P* < 0.05). Although it seemed that subjects with TNs had a greater BMI than in those without TNs, the changes were not significant (*P* > 0.05). For UIE, HDL, triglyceride, and diastolic blood pressure no statistically significant differences between the two groups were observed. Of the five investigated parameters related to thyroid function indexes and autoantibodies, none resulted in statistically significant differences between TN-positive and TN-negative individuals. Of the blood trace elements investigated, no difference was found between the two groups for Mg, Zn, and Pb. It seemed that the levels of Mn in subjects with TNs were marginally higher than in those without TNs (*P* = 0.051). However, levels of copper were significantly higher in individuals with TNs, while their iron levels were lower (*P* < 0.05).

**Table 1:** General characteristic of all subjects of those with and without thyroid nodules (TNs).

**Parameter**	**All**	**TN (–)**	**TN (+)**	***p*-value**
N (%)	1,048 (100%)	505 (48.2%)	543 (51.8%)	-
Age (years)	49.5 ± 14.4	46.0 ± 13.9	52.8 ± 14.1	<0.001
Men (%)	43.7%	54.7%	33.5%	<0.001
Smoking status				0.025
Current smokers (%)	15.7%	18.1%	13.4%	
Ex-smokers (%)	5.4%	6.0%	4.8%	
Non-smokers (%)	78.9%	75.9%	81.8%	
Excessive alcohol consumption (%)	7.2%	9.3%	5.3%	0.031
**Metabolic factors**				
BMI (kg/m^2^)	24.0 ± 3.60	23.8 ± 3.66	24.2 ± 3.54	0.121
HOMA-IR	2.08 (1.29, 3.15)	1.93 (1.17, 3.01)	2.27 (1.38, 3.26)	0.009
HbA1c (%)	5.85 ± 0.922	5.77 ± 0.989	5.93 ± 0.849	<0.001
LDL (mmol/L)	3.24 ± 0.943	3.16 ± 0.996	3.32 ± 0.884	0.007
HDL (mmol/L)	1.41 ± 0.322	1.40 ± 0.325	1.42 ± 0.319	0.549
TG (mmol/L)	1.23 (0.880, 1.83)	1.21 (0.860, 1.85)	1.24 (0.905, 1.83)	0.535
TC (mmol/L)	5.45 ± 1.15	5.34 ± 1.17	5.55 ± 1.13	0.002
Systolic pressure (mm Hg)	131 ± 19.1	129 ± 18.4	132 ± 19.5	0.004
Diastolic pressure (mm Hg)	85.8 ± 12.0	85.3 ± 12.0	86.3 ± 12.0	0.207
**Thyroid hormones**				
TSH (μIU/mL)	2.10 ± 3.57	2.01 ± 2.41	2.19 ± 4.38	0.411
FT3 (pmol/L)	5.53 ± 0.833	5.53 ± 0.716	5.52 ± 0.929	0.783
FT4 (pmol/L)	11.4 ± 1.71	11.4 ± 1.56	11.3 ± 1.85	0.489
TPOAb-positive (%)	16.8%	16.7%	16.8%	0.975
TgAb-positive (%)	10.2%	10.3%	10.1%	0.928
**Trace elements**				
Magnesium (mg/L)	41.8 ± 4.46	41.7 ± 4.56	41.9 ± 4.35	0.351
Manganese (μg/L)	13.2 ± 4.08	12.9 ± 4.08	13.4 ± 4.08	0.051
Iron (mg/L)	506 ± 57.1	513 ± 55.6	499 ± 57.7	<0.001
Copper (μg/L)	887 ± 125	836 ± 118	934 ± 112	<0.001
Zinc (mg/L)	6.33 ± 0.935	6.30 ± 0.909	6.36 ± 0.959	0.284
Lead (μg/L)	20.0 (15.0, 27.0)	20.0 (15.0, 26.0)	21.0 (16.0, 27.0)	0.114
UIE (ug/g)	80.0 (55.5, 116)	78.7 (55.5, 112)	81.3 (55.5, 120)	0.3459

### Association Between Trace Elements and Thyroid Nodules

**Figure 2:**
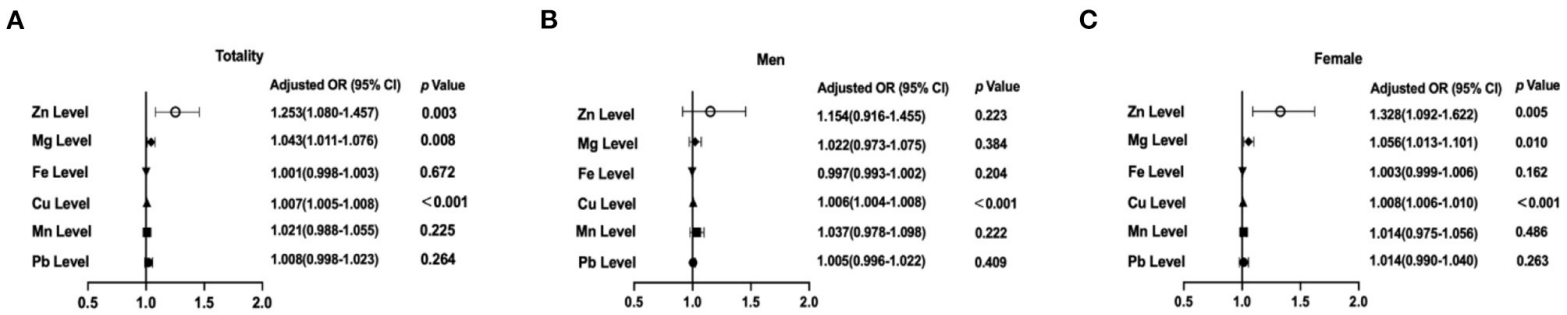
Associations of selected trace elements levels with the prevalence of thyroid nodules (TNs) among the participants. **(A)** Selected trace elements levels and the prevalence of TN in total participants; **(B)** Selected trace elements levels and the prevalence of TNs in males; **(C)** Selected trace elements levels and the prevalence of TNs in females. In the total population, the model was adjusted for model 1. In stratified analysis by sex, the model was adjusted for age, UIE.

**Figure 3:**
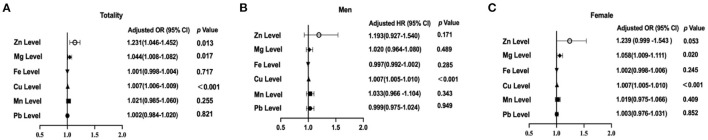
Associations of selected trace elements levels with the prevalence of thyroid nodules (TNs) among the participants. **(A)** Selected trace elements levels and the prevalence of TN in total participants; **(B)** Selected trace elements levels and the prevalence of TNs in males; **(C)** Selected trace elements levels and the prevalence of TNs in females. In the total population, the model was adjusted for model 2. In stratified analysis by sex, the model was adjusted for age, UIE, BMI, smoking status, excessive alcohol consumption, HbA1c, TC, TG, and TSH.

It was assessed whether associations existed between the determined trace element levels and the presence of TNs, for which logistic regression analyses were performed by means of two models. Model 1 merely adjusted for sex, age, and UIE, while model 2 further adjusted for BMI, smoking status, excessive alcohol consumption, three metabolic factors (HbA1c, TC and TG), and TSH. The ORs for TNs across the selected trace elements among all participants are summarized in Table 2. After adjusting for model 1, serum levels of Zn, Mg and Cu were associated with the presence of TNs in the total population (all *P* < 0.05; Figure 2A). The sex-stratified analysis resulted in similar findings about the correlation between Cu level and the presence of TNs after adjusting for age and UIE (*P* < 0.001; Figures 2B,C). After stratification by sex, Zn and Mg levels were still independently associated with the prevalence of TNs in females (*P* < 0.05; Figure 2C). Neither Zn nor Mg levels was significantly associated with the prevalence of TNs in males (*P* > 0.05; Figure 2B). After adjusting for five more factors, as in Model 2, subjects with higher levels of Zn, Mg and Cu had 1.23-fold (95% CI 1.05, 1.45), 1.04–fold (95% CI 1.01, 1.08), and 1.007-fold (95% CI 1.006, 1.009) increased risks of the presence of TNs in the total population (*P* = 0.013, 0.017, and < 0.001, resp.; Figure 3A). In stratified analysis by sex, women with higher levels of Zn, Mg and Cu had 1.24-fold (95% CI 1.00, 1.54), 1.06-fold (95% CI 1.01, 1.11), and 1.007-fold (95% CI 1.005, 1.01) increased risks of the onset of TNs (*P* = 0.053, 0.02, and < 0.001, resp.; Figure 3C); while in males only the association between Cu level and the prevalence of TNs remains significant (*P* < 0.001; Figure 3B).

**Table 2:** Binary logistic analysis between blood trace elements and presence of thyroid nodules.

**Trace elements**	Model 1^*^		Model 2^**^	
	**OR (95%CI)**	***p*-value**	**OR (95%CI)**	***p*-value**
Zinc (mg/L)	1.25 (1.08, 1.46)	0.003	1.23 (1.05, 1.45)	0.013
Magnesium (mg/L)	1.04 (1.01, 1.08)	0.008	1.04 (1.01, 1.08)	0.017
Iron (mg/L)	1.001 (0.998, 1.003)	0.672	1.001 (0.998, 1.004)	0.717
Copper (μg/L)	1.007 (1.005, 1.008)	<0.001	1.007 (1.006, 1.009)	<0.001
Manganese (μg/L)	1.02 (0.99, 1.06)	0.225	1.02 (0.99, 1.06)	0.255
Lead (μg/L)	1.008 (0.998, 1.023)	0.264	1.00 (0.98, 1.02)	0.821

* *Model 1: adjusting for sex, age and UIE*.

** *Model 2: adjusting for sex, age, UIE, BMI, smoking status, excessive alcohol consumption, HbA1c, TC, TG, and TSH*.

### Association Between Trace Elements Levels Quartiles With Presence of Thyroid Nodules

**Figure 4:**
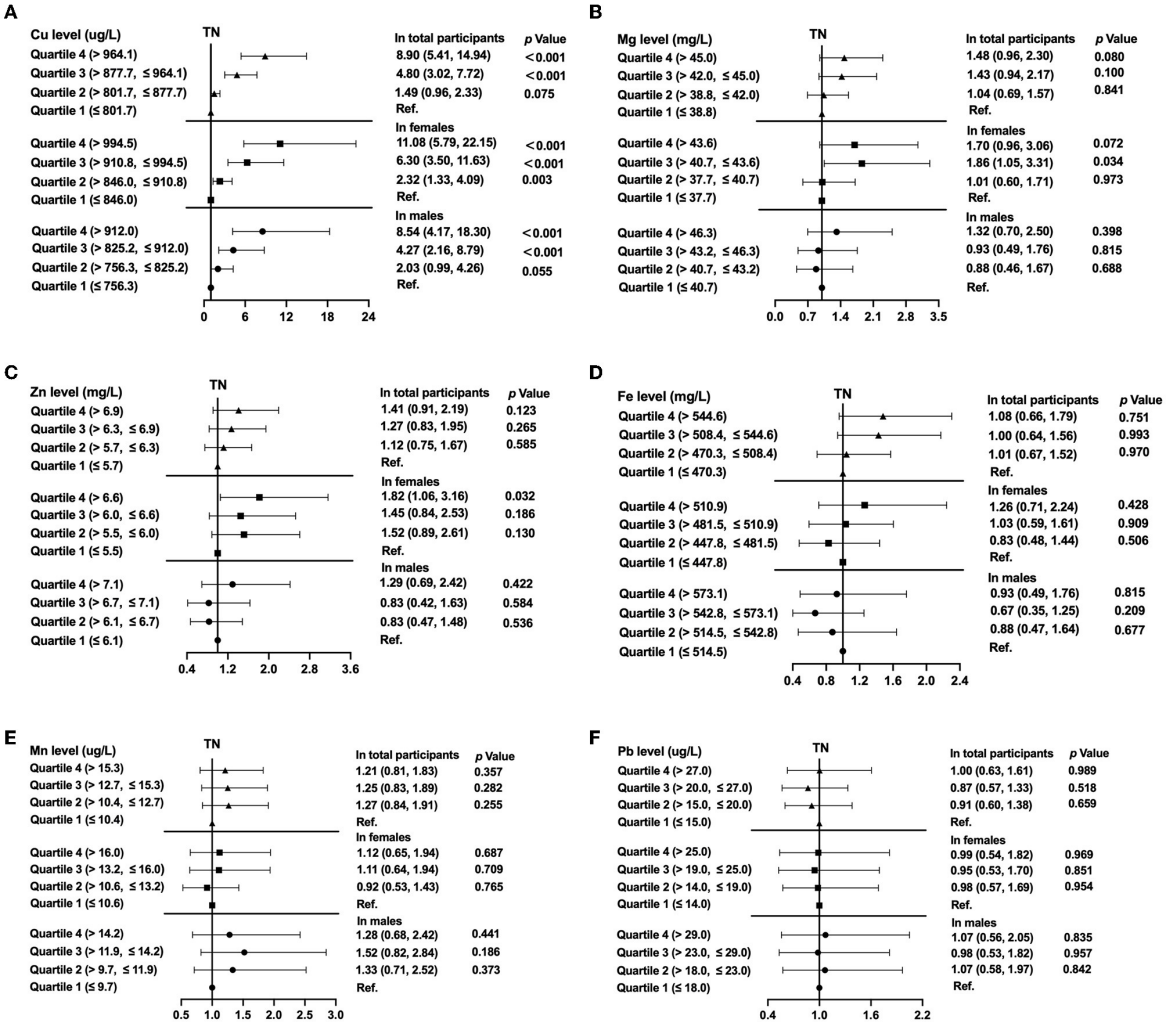
The prevalence of thyroid nodules (TNs) by quartiles of trace element levels, showing odds ratios for Copper **(A)**, Magnesium **(B)**, Zinc **(C)**, Iron **(D)**, Manganese **(E)**, Lead **(F)**. In the total population, the model was adjusted for model 2. In stratified analysis by sex, the model was adjusted for age, UIE, BMI, smoking status, excessive alcohol consumption, HbA1c, TC, TG, and TSH.

Binary logistic regression analysis was also performed for the quartiles of the selected trace elements Zn, Mg, Fe, Cu, Mn, or Pb and their association with presence of TNs both in the total population and in stratified analysis by sex after adjusting for potential confounders (Figure 4). This clearly demonstrated that the level of copper determined in the blood was significantly associated with the presence of thyroid nodules among all participants based on model 2 (*P* < 0.01); and the RCS analysis revealed a non-linear relationship between Cu levels and TNs (*P* for non-linearity = 0.0004; Supplementary Figure 1A). For the highest compared with the lowest quartile of copper level, the OR of the TNs was 8.90-fold (95%CI 5.41, 14.94, *P* < 0.001; Figure 4A). In stratified analysis by sex, men in the fourth quartile of Cu level had a 8.54-fold (95%CI 4.17, 18.30) risk of the presence of TNs and women in the fourth quartile of Cu level had a 11.08-fold (95%CI 5.79, 22.15) risk of the presence of TNs after adjusting for age, UIE, BMI, smoking status, excessive alcohol consumption, HbA1c, TC, TG and TSH (Both *P* < 0.001; Figure 4A). Women in the third and fourth quartile of magnesium level had marginal increased risks of the onset of TNs (OR = 1.86, 95% CI 1.05, 3.31, *P* = 0.034, OR = 1.70, 95% CI 0.96, 3.06, *P* = 0.072, resp.; Figure 4B). Subjects in the highest quartile of zinc level had a 1.82-fold (95%CI 1.06, 3.16) risk of the prevalence of TNs in females (*P* = 0.032; Figure 4C). No association between iron, manganese, or lead quartiles and TNs was found (Figures 4D–F). In addition, the non-linear association between Mn, Fe or Pb and the prevalence of TNs was not detected in the restricted cubic spline model (*P* for non-linearity > 0.05; Supplementary Figures 1D–F).

## Discussion

According to data from the American Association of Clinical Endocrinologists (AACE), the American College of Endocrinology (ACE) and the Italian Associazione Medici Endocrinologi (AME), thyroid nodules can be detected in up to 60% of healthy individuals (5). The prevalence of TNs reported here reached 51.8%, which is in good agreement with reported prevalence. It has been described that TNs are more common in the elderly and in females and that they are also frequently associated with obesity, insulin resistance and dyslipidemia (5, 24–26). Indeed, we found TNs were more common in women and in older age groups. Moreover, subjects with TNs had higher systolic blood pressure and we recorded higher levels of HOMA-IR, HbA1c, LDL, and total cholesterol. These findings are in line with what has been previously reported, although a correlation with increased BMI could not be significantly demonstrated in our dataset.

Human exposure to trace elements can occur in various ways and is not necessarily harmful, depending on the dose (27). Five of the selected trace elements determined here are needed for normal cellular processes as nutritional factors, with the exception of lead, which is toxic even at low doses. So far, evidence was lacking whether trace element levels correlated with presence of TNs, and this was the main subject of this study. Our results revealed significant differences in blood levels of trace elements between TN-positive and TN-negative subjects, in particular for copper, magnesium, zinc and iron: participants with TNs had higher levels of these trace metals in their blood, but lower levels of iron. We then evaluated the adjusted ORs for TN. After correction for sex, age and UIE, higher levels of Cu, Mg, and Zn were correlated with higher prevalence of TNs among all participants. Even after further correction for smoking status, alcohol overconsumption, glycated hemoglobin, total cholesterol, triglycerides and TSH, we observed that subjects with higher concentrations of copper, zinc and magnesium had significantly greater risks of TNs among all participants. The quartile analysis in the total population enforced the findings, as the 25% of subjects with the highest amounts of copper and produced the highest OR for presence of TNs, and the results remained consistent in the stratified analysis by sex. After stratification by gender, higher quartiles of magnesium and zinc levels had greater risks of TNs in females.

Copper can act as an antioxidant or an oxidant, and it is a cofactor of numerous enzymes including cytochrome C, superoxide dismutase (SOD), and lysyl oxidase. A correlation between thyroid function and blood Cu levels has been observed before, as patients with thyroid cancer were reported to have elevated serum Cu levels compared to healthy controls (19). Following radioactive exposure from the Chernobyl disaster, thyroid tissue from patients were shown to have decreased Cu concentrations compared to controls (28). Higher copper concentrations were detected in plasma from patients with nodular goiter (29) and in serum from individuals with diffuse goiter (30). Kosova et al. reported increased serum Cu levels in malignant thyroid patients and decreased levels in benign cases (31). However, the relationship between copper and thyroid nodules is uncharted. Here we report that the higher quartiles of copper were associated with a greater risk of TNs: a 8.90-fold increase in the risk of TNs was observed in participants of the highest quartile compared with the lowest quartile for copper levels among all participants, which confirmed a strong positive association between blood copper levels and TNs risk. The MAPK signaling pathway, which is involved in cellular proliferation, is stimulated by copper, and copper cellular influx also enhances phosphorylation of ERK1/2 through interaction of Cu with MEK1 (32). We hypothesized that higher Cu levels are involved in the pathogenesis of TNs by Cu-MEK1 interaction.

At present, there is limited data on the role of magnesium in thyroid metabolism. Magnesium might antagonize the effect of thyroxine on recipient cells (33). Long-term supplementation of magnesium may normalize thyroid morphology in patients with thyroid disease (34). A meta-analysis reported that subjects with thyroid cancer had lower Mg levels in their serum (19). Kravchenko et al. showed that median concentrations of magnesium were elevated in patients with nodular goiter compared to controls (20). Our study found that subjects in the third and fourth quartile of magnesium levels had marginal increased risks of the onset of TNs compared with those in the other quartile among women. Magnesium supplementation should be carried out cautiously in females.

Zinc plays critical roles in growth, sexual maturation, immune function, wound healing, and other processes (29). Nuclear T3 receptor is probably a zinc-binding protein, which indicates an association exists between zinc and thyroid metabolism, and thyroid hormones affect zinc absorption (35). Moreover, zinc deficiency can lead to hypothyroidism (36). The association between zinc intake via food and TN development is yet to be established. Meh et al. detected lower Zn concentration in serum of patients with goiter compared to controls (12), but in another study no statistically significant association between zinc levels and nodular goiter was observed (37). In our study, the positive association between zinc concentrations and TNs was noted, also subjects in the highest quartile of zinc level had a higher risk of the prevalence of TNs in females. Zinc is an essential nutrient for good health, but an excess can be harmful, especially for women.

As the most abundant transition metal in the human body (38), iron is an element critical for normal growth and development (39). Iron homeostasis strongly correlates with the thyroid function. Active T3 has been proved to regulate hepatic ferritin expression, and insufficiency of iron affects production of thyroid hormones since heme-dependent enzyme TPO decreases (21). Kravchenko et al. (20) and Kazi et al. (30) observed lower Fe concentration in serum of patients with nodular goiter in comparison with controls. We observed decreased concentration of iron in serum for the participants with TNs in comparison with those without TNs, which might suggest that iron deficiency is related to thyroid nodule. Manganese is a fundamental element involved in many physiological processes in mammals. Manganese may affect the thyroid homeostasis via direct or indirect mechanisms (40). It is worth highlighting that exposure to excessive manganese is potentially toxic (40). Liu et al. suggested that the levels of Mn showed significant negative association with the risk of thyroid tumor and goiter (41). We observed that the levels of Mn in subjects with TNs were marginally higher than in those without TNs. Lead is a widespread environmental toxicant (42). The mechanism of toxic effects of Pb on thyroid gland is still unidentified. As a consequence of prolonged exposure to the high content of Pb in the thyroid tissue, dysfunction of thyroid follicular cells occurs; while the fact of an inconsistency of results between the associations of Pb with thyroid hormones should be highlighted (21). No statistically significant association was found between Pb and TNs in this study. More research is required to determine whether lead or manganese is associated with the presence of thyroid nodules.

The strengths of our study are a relatively large sample size, inclusion of multiple trace element analyses, and involvement of healthy subjects living in the iodine-adequate area. All included subjects were enrolled from the local community, so that results may be more representative of the general population than studies conducted with a clinical population. The methodology involved questionnaires and anthropometric measurements that were completed by trained researchers, and thyroid ultrasound examinations were performed by highly qualified medical doctors subject to strong quality control standards.

However, there were also limitations in our study. Due to the nature of this cross-section study, it is difficult to provide data on causality, as only associations are being assessed. For causality assessment, a longitudinal study would be required. Our study population was restricted to one ethnic group, Han Chinese, which leaves the possibility that the results may not be generally applicable to other ethnicities. Furthermore, FNAB is regarded as the gold standard to diagnosis to distinguish benign and malignant and identify the nature of the thyroid nodules. The relationship between trace elements and benign or malignant nodules cannot be evaluated, which needs to be further explored.

In conclusion, we report that TNs are more prevalent in females in the iodine-adequate area of Guangdong, China. In our study, the imbalance of selected trace elements (copper, magnesium and zinc) in the body is related to the presence of TN among healthy subjects. The data from this study suggested that detection of copper element may be of considerable significance for the clinical management of thyroid nodules and whether it is suitable to intake multivitamins and minerals supplementation should be cautiously considered. However, whether copper plays a role in the pathogenesis of TN remains to be elucidated. Further prospective, larger studies with long-term follow-up periods are warranted.

## Data Availability Statement

The original contributions presented in the study are included in the article/Supplementary Material, further inquiries can be directed to the corresponding authors.

## Ethics Statement

The studies involving human participants were reviewed and approved by Shunde Hospital of Southern Medical University (The First People's Hospital of Shunde District, Foshan City). The patients/participants provided their written informed consent to participate in this study.

## Author Contributions

JS and HW designed the study, revised the manuscript, and supervised the study. HZ undertook the statistical analysis and wrote and revised the manuscript. YH checked the results and revised the manuscript. YLu contributed to the discussion. All authors participated in data acquisition. All authors reviewed and approved the final manuscript for publication.

## Funding

This study was supported by the Funding for the construction of demonstration zones for the comprehensive prevention and control of chronic non-communicable diseases in Shunde District; the National Natural Science Foundation of China (82170800); Guangdong Basic and Applied Basic Research Foundation (2021A1515110682); and Research initiation Project of Shunde Hospital of Southern Medical University (SRSP2021001).

## Conflict of Interest

The authors declare that the research was conducted in the absence of any commercial or financial relationships that could be construed as a potential conflict of interest.

## Publisher's Note

All claims expressed in this article are solely those of the authors and do not necessarily represent those of their affiliated organizations, or those of the publisher, the editors and the reviewers. Any product that may be evaluated in this article, or claim that may be made by its manufacturer, is not guaranteed or endorsed by the publisher.
