# Supplementary material for: The Association of Thyroid Nodules With Blood Trace Elements Identified in a Cross-Section Study
Source: Front Nutr. 2022 Apr 28;9:870873. doi: 10.3389/fnut.2022.870873 (PMC9096353; doi:10.3389/fnut.2022.870873)
Supplement: Supplementary file 1 [file Data_Sheet_1.docx]

**SUPPLEMENTARY MATERIAL**

**
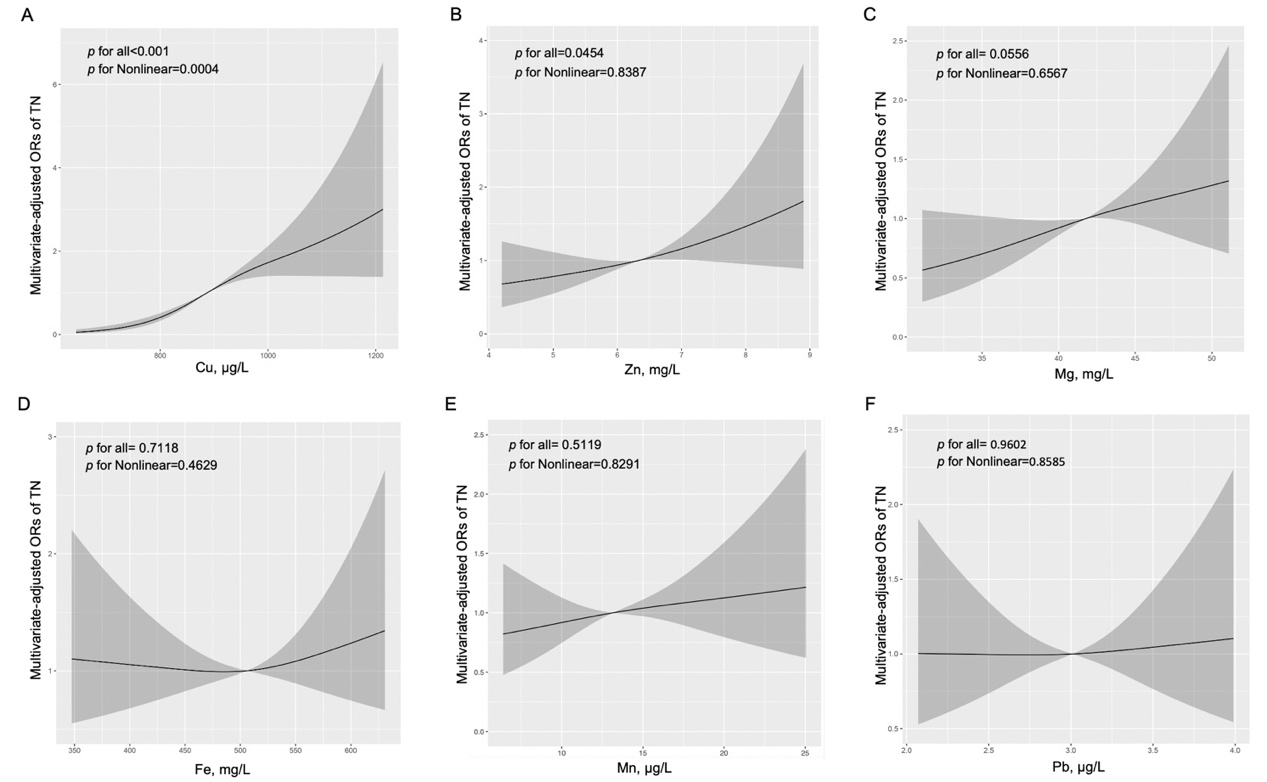
**

**Supplementary Figure 1** The non-linear analysis between selected trace elements and the prevalence of TN among the participants. (A) Copper and TNs; (B) Zinc and TNs; (C) Magnesium and TNs; (D) Iron and TNs; (E) Manganese and TNs; (F) Lead and TNs. The restricted cubic spline analysis was based on Model 2.

**Supplementary Table 1** The prevalence of TNs by quartiles of selected trace elements

| Trace elements | Prevalence of TNs (%) | | | | p-value |
| --- | --- | --- | --- | --- | --- |
|  | Quartile 1 | Quartile 2 | Quartile 3 | Quartile 4 |  |
| Zinc (mg/L) | ﻿50.2% | ﻿52.0% | 53.3% | 52.3% | 0.908 |
| Magnesium (mg/L) | 49.8% | 50.8% | 56.2% | 50.8% | 0.444 |
| Iron (mg/L) | 59.4% | 56.2% | 48.8% | 43.3% | <0.001 |
| Copper (µg/L) | 23.1% | 37.4% | 65.8% | 79.8% | <0.001 |
| Manganese (µg/L) | 48.2% | 51.0% | 52.9% | 55.9% | 0.344 |
| Lead (µg/L) | 48.5% | 51.4% | 52.9% | 55.1% | 0.492 |

**Supplementary Table 2** The blood trace elements in TN-positive subjects with the thyroid US characteristics ﻿associated with malignancy

| Trace elements | Thyroid US characteristics | | | | | | | | |
| --- | --- | --- | --- | --- | --- | --- | --- | --- | --- |
|  | Diameter⩽1cm | ﻿Diameter＞1cm | *p*-value | Solitary | Multiple | *p*-value | No calcification | Calcification | *p*-value |
| N (%) | 383 | 160 | - | 246 | 297 | - | 469 | 74 | - |
| Zinc (mg/L) | 6.44±0.998 | 6.18±0.833 | 0.002 | 6.44±0.974 | 6.29±0.943 | 0.068 | 6.37±0.964 | 6.30±0.928 | 0.575 |
| Magnesium (mg/L) | 42.1±4.30 | 41.5±4.48 | 0.153 | 41.7±4.11 | 42.0±4.55 | 0.415 | 41.9±4.32 | 42.2±4.57 | 0.538 |
| Iron (mg/L) | 501±58.5 | 495±55.9 | 0.261 | 502±59.4 | 497±56.2 | 0.284 | 499±58.7 | 498±51.6 | 0.776 |
| Copper (µg/L) | 936±114 | 929±110 | 0.505 | 933±106 | 936±117 | 0.779 | 936±114 | 922±101 | 0.287 |
| Manganese (µg/L) | 13.5±4.08 | 13.1±4.05 | 0.307 | 13.3±4.00 | 13.5±4.14 | 0.516 | 13.4±4.16 | 13.2±3.52 | 0.585 |
| Lead (µg/L) | 21.0 (15.0,28.0) | 21.0 (16.0,26.0) | 0.951 | 20.0 (15.0,26.0) | 21.0 (16.0,28.0) | 0.033 | 21.0 (15.8,27.3) | 21.0 (17.0,27.0) | 0.692 |

**Supplementary Table 3** Binary logistic analysis between blood trace elements and the thyroid US characteristics ﻿associated with malignancy

| Trace elements | Diameter＞1cm | | Multiple | | Calcification | |
| --- | --- | --- | --- | --- | --- | --- |
|  | OR (95%CI) | *p*-value | OR (95%CI) | *p*-value | OR (95%CI) | *p*-value |
| Zinc (mg/L) | 0.819 (0.644, 1.036) | 0.099 | 0.856 (0.683, 1.068) | 0.171 | 0.962 (0.710, 1.295) | 0.798 |
| Magnesium (mg/L) | 0.961 (0.913, 1.012) | 0.134 | 1.021 (0.973, 1.072) | 0.403 | 0.998 (0.934, 1.067) | 0.955 |
| Iron (mg/L) | 0.999 (0.995, 1.003) | 0.708 | 1.000 (0.996, 1.004) | 0.845 | 0.999 (0.994, 1.005) | 0.827 |
| Copper (µg/L) | 0.998 (0.996, 1.000) | 0.055 | 0.998 (0.996, 1.000) | 0.113 | 0.998 (0.995, 1.001) | 0.244 |
| Manganese (µg/L) | 0.992 (0.939, 1.046) | 0.764 | 1.037 (0.986, 1.091) | 0.162 | 0.994 (0.924, 1.065) | 0.867 |
| Lead (µg/L) | 1.008(0.998,1.023) | 0.264 | 1.004 (0.979, 1.029) | 0.767 | 0.980 (0.946, 1.012) | 0.243 |
